# Genetically engineered bacteria as inflammatory bowel disease therapeutics

**DOI:** 10.1016/j.engmic.2024.100167

**Published:** 2024-09-01

**Authors:** Zhen-Ping Zou, Xiao-Peng Zhang, Qian Zhang, Bin-Cheng Yin, Ying Zhou, Bang-Ce Ye

**Affiliations:** Laboratory of Biosystems and Microanalysis, State Key Laboratory of Bioreactor Engineering, East China University of Science and Technology, Shanghai, 200237, China

**Keywords:** Genetically engineered bacteria, Inflammatory bowel disease, Bacterial therapeutics

## Abstract

Inflammatory bowel disease (IBD) is a chronic and recurrent disease caused by immune response disorders that disrupt the intestinal lumen symbiotic ecosystem and dysregulate mucosal immune functions. Current therapies available for IBD primarily focus on symptom management, making early diagnosis and prompt intervention challenging. The development of genetically engineered bacteria using synthetic biology presents a new strategy for addressing these challenges. In this review, we present recent breakthroughs in the field of engineered bacteria for the treatment and detection of IBD and describe how bacteria can be genetically modified to produce therapeutic molecules or execute diagnostic functions. In particular, we discuss the challenges faced in translating live bacterial therapeutics from bacterial design to delivery strategies for further clinical applications.

## Highlights

• Bacteria can be genetically modified to produce therapeutic molecules or execute diagnostic functions
• Live medicines based on the bacterial chassis represent a breakthrough therapeutic strategy that leverages their ability for mass production and ease of genetic manipulation
• Several bacterial sensors have been developed to detect IBD biomarkers
• Before clinical translation, it is essential to assess the safety, efficacy, biocontainment, and stability of bacterial therapies

## Graphical abstract

**Figure:**
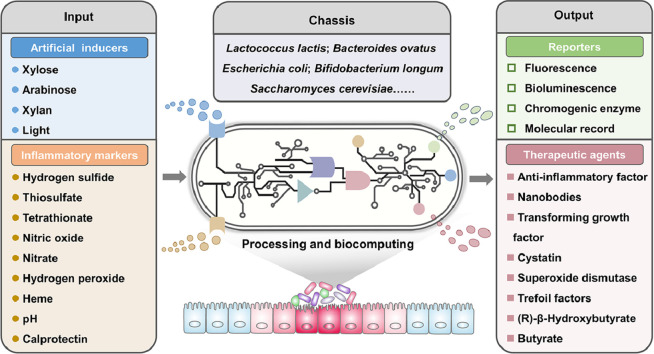

## 1 Introduction

Inflammatory bowel disease (IBD) is characterized by chronic intestinal inflammation involving the ileum, colon, and rectum, and includes ulcerative colitis, Crohn's disease, and IBD unclassified [[1], [2], [3]]. IBD is a significant healthcare concern owing to its increasing incidence and global prevalence, and can ultimately lead to cancer and life-threatening complications [4]. Conventional therapeutics for IBD treatment include aminosalicylic acid formulations, glucocorticoids, and immunosuppressants (infliximab and adalimumab) [5]. However, their high cost, uncertain safety, and likelihood of recurrence significantly restrict their clinical applications [5,6]. Moreover, early diagnosis of IBD contributes to early intervention and symptom control [7,8]. Current IBD diagnostic methods include endoscopy [9,10], which is invasive and patients are often unwilling to frequently track disease progression, and analysis of inflammatory biomarkers (e.g., calprotectin and lactoferrin), which may not accurately reflect native conditions [[11], [12], [13]]. Moreover, many inflammatory biomarkers, such as reactive oxygen species (ROS), reactive nitrogen species, and hydrogen sulfide (H_2_S) are highly reactive and have short half-lives in the intestine, which makes them difficult to detect in the stool [[14], [15], [16], [17]]. Consequently, there is an urgent need to develop new treatments and detection strategies for IBD.

**Fig. 1:**
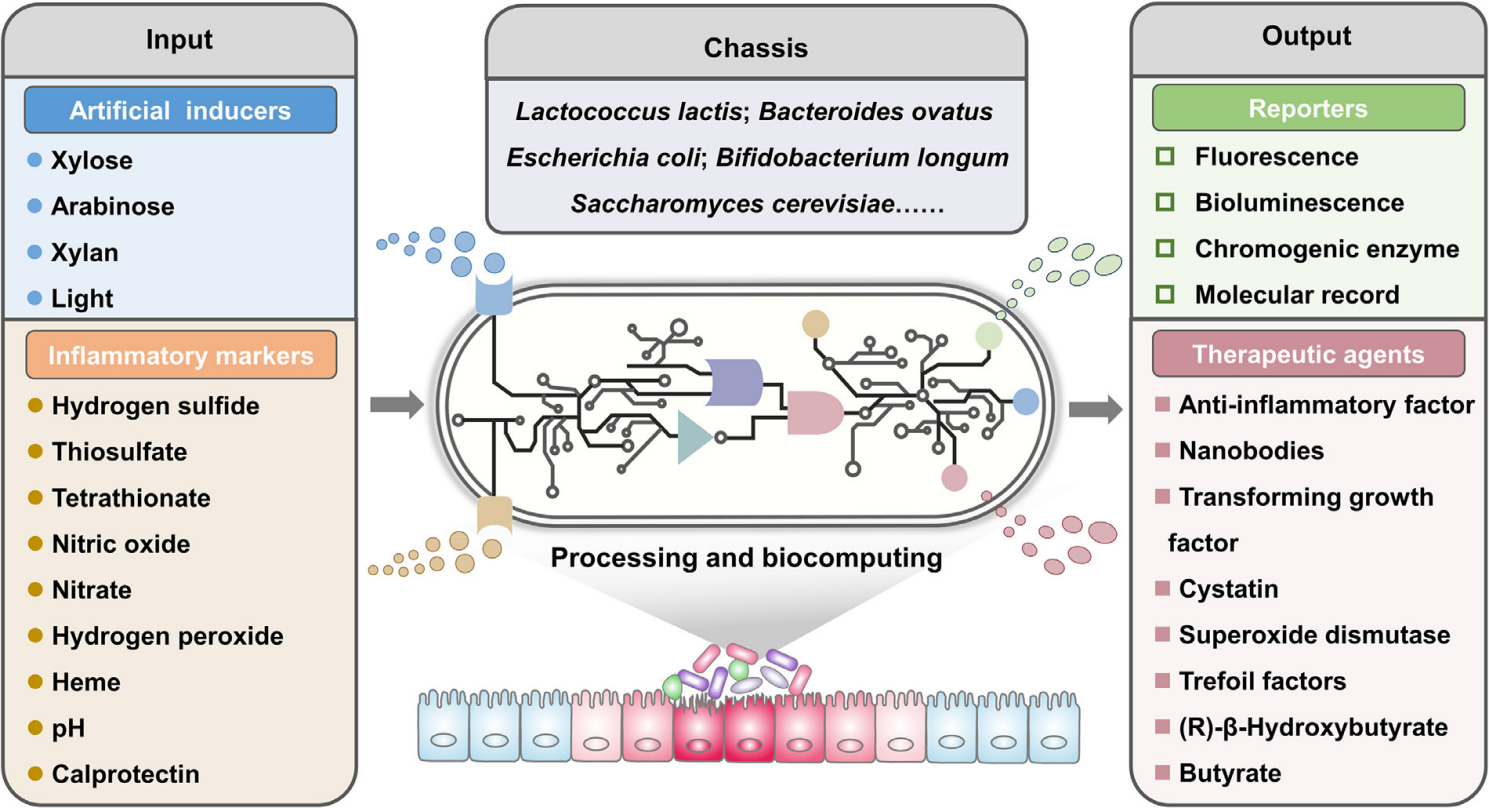
Schematic of genetically engineered bacteria for IBD management. Central to these bacteria is a chassis integrated with a sophisticated genetic circuit, tasked with signal detection and response. The genetic circuit is segmented into three modules: a sensing module, a signal processing module, and an output module. The sensing module detects both artificially provided signals and disease biomarkers. Upon signal acquisition, bio-computational processing occurs, leading to the activation of the output module. This final module is engineered for adaptability, capable of producing a variety of either detectable reporter signals or targeted therapeutic agents.

Probiotics, with their inherent properties in the intestine, have been proposed as promising solutions to improve intestinal health and provide benefits [[18], [19], [20]]. Nevertheless, limited by their uncontrollable physiological behavior and unsatisfactory therapeutic function, these wild-type strains cannot effectively treat diseases [21,22]. The development of synthetic biology tools enables the convenient engineering of bacteria to enhance their therapeutic efficacy and safety by delivering therapeutic payloads *in situ* [[23], [24], [25], [26], [27], [28], [29], [30]]. Importantly, some intelligently engineered bacteria have recently been designed for personalized medicine, including inflammatory-marker-driven feedback control of drug release systems and multiple function integrated systems [31,32] (Fig. 1). In this review, we summarize the recent advances in bacterial engineering for the treatment and detection of IBD, highlighting how engineered bacteria can produce therapeutic molecules or execute diagnostic functions. In particular, we discuss several important challenges faced by genetically engineered bacteria in the diagnosis and treatment of IBD before clinical translation, such as biocontainment, accurate responses, targeting, and delivery strategies.

## 2 Genetically engineered bacteria for the treatment of IBD

### 2.1 Constitutive system

Live medicines based on the bacterial chassis represent a breakthrough therapeutic strategy that leverages their ability for mass production and ease of genetic manipulation. Compared with traditional oral or systemic therapies, *in vivo* production and delivery of therapeutics through engineered microbes offers several advantages, including lower production costs and non-invasiveness [33,34]. Constitutive systems are commonly employed for the expression of therapeutic agents in bacteria. A constitutive expression system in bacteria features a promoter that remains perpetually active, resulting in the uninterrupted expression of the target gene, irrespective of external conditions. The promoter in this system is known as a constitutive promoter because it continuously initiates transcription without the need for specific inducers or environmental conditions. This simplicity makes constitutive expression systems a popular choice for using bacteria to produce therapeutic agents.

Early attempts mainly focused on synthesizing anti-inflammatory factors using lactic acid bacteria (LAB) as a chassis [35]. LAB have long-term safety records and are widely used in fermented foods. *Lactobacillus* and *Lactococcus* are the most widely studied LAB and are used either as producer cells or as vectors for the delivery of therapeutic molecules for the prevention and treatment of different medical conditions [36]. As early as 2000, Steidler *et al*. developed a *Lactococcus lactis* strain to secret murine IL-10 (an anti-inflammatory cytokine) and demonstrated that administration of this engineered *L. lactis* caused a 50% reduction in dextran sulfate sodium (DSS) induced colitis in mice and prevented the onset of colitis in IL-10^−^/^−^ mice [37]. Importantly, the effective amount of IL-10 secreted by engineered *L. lactis* was estimated to be several orders of magnitude lower than that secreted by conventional systemic administration. The ability of engineered *L. lactis* to express and secrete IL-10 (fused with the Usp45 secretion peptide) has also been investigated [38]. Furthermore, Steidler *et al*. replaced the thymidylate synthase gene *thyA* of *L. lactis* with a human *IL-10*-encoding gene (LL-Thy12) to prevent engineered bacteria from accumulating in the intestine and improve biosafety [39]. Phase 1 clinical trials have shown that LL-Thy12 is safe and effective in patients with Crohn's disease [40]; however, an insufficient therapeutic effect was found in a phase 2a clinical study (Identifier: NCT00729872). Other immunosuppressive cytokines have also been expressed in LAB chassis to treat IBD. Engineered *L. lactis* (LL-IL-27) that expresses and secretes IL-27 reduced colitis by increasing IL-10 production [41]. Notably, the LL-IL-27 strain demonstrated superior efficacy in treating inflammation compared to LL-IL-10, as it can induce higher levels of IL-10 in the gastrointestinal tract. *Escherichia coli* Nissle 1917 (EcN), a genetically tractable chassis with Generally Recognized As Safe (GRAS) status, is emerging as a favored carrier for payload delivery [[42], [43], [44]]. EcN has inherent anti-inflammatory and intestinal flora regulatory characteristics and is marketed as the main component of Mutaflor® for the treatment of IBD [[45], [46], [47]]. EcN strains engineered to express IL-10 have been developed and have been shown to effectively alleviate colitis in mouse models [48]. Moreover, oral *E. coli* expressing IL-35 also ameliorated colitis in mice [49]. Considering the differences in the protein expression and secretion systems between *E. coli* and LAB, there is significant variation in the levels and activity of therapeutic agents like IL-10 produced based on these two chassis. Preliminary studies by Gardlik *et. al* indicate that under identical culturing conditions, LAB can secrete higher levels of IL-10 [48]. However, further research is required to investigate the differences in therapeutic agent expression and secretion between these two platforms.

Inhibition of the inflammatory pathway has proven to be an effective strategy for the treatment of IBD [[50], [51], [52]]. Tumor necrosis factor-alpha (TNF-α) is widely considered to be a key factor contributing to the manifestation of various clinical symptoms in IBD [53]. Anti-TNF-α monoclonal antibodies such as infliximab and adalimumab have been used to block TNF-α activity and treat IBD [[54], [55], [56]]. A unique class of immunoglobulins lacking light chains, known as nanobodies (Nbs) or variable heavy chains (VHHs), has been identified in sharks and camels [57,58] and Coppieters *et al.* developed anti-TNF single-domain antibody fragments derived from heavy-chain camelid antibodies [59]. These Nbs can be cloned and easily expressed as recombinant proteins in a bacterial chassis [60]. Leveraging these advantages, Vandenbroucke *et al*. engineered an *L. lactis* strain capable of constitutively secreting monovalent and bivalent murine TNF-Nbs [61]. Daily oral administration of this engineered bacteria significantly reduced colitis in mice. Lynch *et al*. developed a system called PROT_3_EcT, a suite of *E. coli* strains engineered to secrete proteins directly into their surroundings [62]. This platform consists of three components: a modified secretion system (derived from the type III secretion system of *Shigella*), a regulatable transcriptional activator, and a secreted therapeutic payload. PROT_3_EcT can efficiently secrete functional TNF-α Nbs and stably colonize the intestines of mice. A single prophylactic dose of PROT_3_EcT was sufficient to prevent inflammation in a TNBS (2,4,6-trinitrobenzenesulfonic acid) induced colitis model. Helminth cysteine protease inhibitors, such as AvCystatin, can reduce inflammatory diseases via the modulation of macrophages [63,64], and an engineered EcN strain secreting AvCystatin can treat gut inflammation [65]. Wang *et al*. reported that EcN-Sj16, which expresses schistosome immunoregulatory protein (Sj16), a parasite-derived immunoregulatory molecule, can ameliorate IBD in humans [66]. The Sj16 peptide-coding sequence was connected with the α-hemolysin (HlyA) C-terminal signal sequence and this secretion system carried the HlyB-HlyD complex gene sequences that can form membrane pores and recognize the C-terminal protein of HlyA, leading Sj16 to be directly secreted into the extracellular environment. EcN-Sj16 significantly alleviated colitis in mice through the *Ruminococcaceae*/butyrate/retinoic acid axis [67]. Moreover, the protective and therapeutic potentials of trefoil factors (TFF)-expressing *L. lactis* have also been evaluated in DSS-induced colitis model and in IL-10^−^/^−^ mice [68]. TFF molecules exhibit cytoprotective properties, facilitate the healing of epithelial wounds, and contribute to the restoration of the gastrointestinal tract [69]. Most current research focuses on generating a single therapeutic protein, which can potentially result in drug resistance and reduced efficacy over extended periods of usage. Cocktail therapy with multiple drug proteins has been demonstrated to be an effective treatment for IBD [[70], [71], [72]]. A dual bacterial system capable of sustainably producing both the anti-inflammatory cytokine IL-10 and TNF-α nanobody was recently developed. This approach enables the simultaneous neutralization of pro-inflammatory factors and enhancement of the anti-inflammatory pathway. Administration of this cocktail effectively alleviated inflammation, surpassing the results achieved by administering either one of the engineered strains or wild-type EcN alone [73].

Gut microbiota-derived metabolites also play a crucial role in IBD development [74,75]. Synthetic biology strategies to genetically engineer bacteria for sustained metabolite production have been developed as a potential method for the treatment of colitis in mice. (R)-β-hydroxybutyrate (3HB) can serve as an energy source during periods of starvation or exercise and as a therapeutic agent for various diseases [76,77]. Yan *et al*. engineered an EcN strain to synthesize 3HB for sustainable production in the gut of colitis mice and to treat colitis in mice by improving the colonic microenvironment. The engineered EcN strain substantially boosts the levels of gut 3HB and short-chain fatty acids (SCFAs), enhancing them by 8.7-fold and 3.1-fold respectively, compared to the wild-type EcN. The sustained presence of 3HB in the mouse gut encourages the growth of probiotic bacteria, especially *Akkermansia* spp., which increased from an initial 2% to over 31% of the entire microbiome [78]. Several studies have investigated the correlation between butyrate levels and intestinal inflammation [[79], [80], [81]]. Gong *et al*. constructed a butyrate producing EcN strain which protected against colitis and preserved intestinal mucosal homeostasis in mice [82]. The authors demonstrated that butyrate in the gut serves as a protective agent against colitis and enhances the stability of the intestinal mucosa by downregulating gasdermin D, an essential effector of pyroptosis in the colonic epithelium, via its function as an inhibitor of histone deacetylase 3. Recently, Wu *et al*. customized a series of engineered yeasts to produce different concentrations of butyrate for the cell-dependent treatment of IBD [83]. Metabolic disorders of various compounds such as short-chain fatty acids, bile acids, and tryptophan are closely related to the occurrence and development of IBD [74,75]. For example, some tryptophan metabolites, indole-3-lactic acid, indole-3-propionic acid, and indole-3-acetic acid have been demonstrated to effectively reduce intestinal inflammation and regulate the gut microbiota in boss DSS-induced and IL-10^−/−^ spontaneous colitis models [84]. Extracellular adenosine triphosphate signals produced by the gut microbiota and host cells activate purinergic signaling to promote the production of pro-inflammatory cytokines and the activation of effector T cells, while inhibiting regulatory T cell responses, thereby promoting intestinal inflammation [85,86]. Developing live bacteria capable of producing or degrading these compounds holds great promise for enhancing the treatment of IBD.

Many studies have indicated that colitis is associated with increased ROS [[87], [88], [89]]. ROS are neutralized by superoxide dismutase (SOD), which plays a key role in colitis. *Bifidobacterium longum* [90] and EcN [91] that express SOD have been used to suppress and treat IBD, respectively. Enzyme proteins [92] or peptides [93,94] with anti-inflammatory properties expressed by bacteria have also shown significant protective effects against colitis in mice. Engineered bacteria with a constitutive system for IBD treatment are summarized in Table 1.

**Table 1:** Summary of engineered bacteria using a constitutive system for IBD treatment.

Chassis	Payload	Model	Refs
*Lactococcus lactis*	Murine IL-10	DSS-induced colitis and IL-10^−^/^−^ mice	[37]
*Lactococcus lactis*	Human IL-10	Ileal loop of a pig	[39]
*Lactococcus lactis*	Murine IL-27	Rag^−^/^−^ mice	[41]
*Lactococcus lactis*	Monovalent and bivalent murine (m)TNF-neutralizing Nanobodies	IL10^−^/^−^ mice	[61]
*Lactococcus lactis*	Trefoil factors	DSS-induced colitis and IL-10^−^/^−^ mice	[68]
*Lactococcus lactis*	Heme oxygenase-1	DSS-induced colitis	[92]
*Escherichia coli* Nissle 1917	Murine IL-10	DSS-induced colitis	[48]
*Escherichia coli* Nissle 1917	IL-35	DSS-induced colitis	[49]
*Escherichia coli* Nissle 1917	TNF-α-neutralizing Nanobodies	DSS-induced colitis and TNBS-induced colitis	[62]
*Escherichia coli* Nissle 1917	AvCystatin	DSS-induced colitis and piglets	[65]
*Escherichia coli* Nissle 1917	Sj16	DSS-induced colitis	[67]
*Escherichia coli* Nissle 1917	Catalase and superoxide dismutase	DSS-induced colitis	[91]
*Escherichia coli* Nissle 1917	(R)-β-Hydroxybutyrate	DSS-induced colitis	[78]
*Escherichia coli* Nissle 1917	Butyrate	DSS-induced colitis and GSDMD−/− mice	[82]
*Saccharomyces cerevisiae*	Butyrate	TNBS-induced enteritis	[83]
*Bifidobacterium longum*	Human manganese superoxide dismutase	DSS-induced colitis	[90]

### 2.2 Artificial intervention system

Although engineered bacteria with constitutively expressed therapeutic payloads have shown promise as potential therapies for gut inflammation, it is important to consider that sustained drug release can place a significant burden on bacteria and may have adverse effects on the host. Compared with constitutive systems, an artificial intervention strategy is an improved choice (Table 2). Artificial intervention expression systems allow for controlled gene expression. The system requires specific inducing molecules provided to initiate transcription promoters. The presence of artificially provided inducers activates the promoter, which then starts the transcription of the gene of interest. Chemical inducers and physical methods are commonly employed in artificial induction systems to regulate cell-specific behavior. TFFs can promote intestinal barrier function and epithelial restitution. Praveschotinunt *et al*. engineered an EcN strain to secrete curli-fused TFFs, which was induced by arabinose. This system is known as the probiotic-associated therapeutic curli hybrid (PATCH) [95]. PATCH ameliorated DSS-induced colitis by healing the mucosa and modulating immunity. Hamady *et al*. devised a bacterial drug delivery system controlled by dietary xylan by modifying the human commensal gut bacterium *Bacteroides ovatus* to produce human growth factors [96] and transforming growth factor β1 [97]. In DSS-treated mice, administration of xylan and engineered bacteria demonstrated significant therapeutic effects. The xylose-inducible expression system (XIES) is an important system [98]. Carmen *et al*. genetically engineered an *L. lactis* strain to produce IL-10 in response to XIES xylose, which effectively prevented IBD in a murine model [99]. A xylose-induced system, including the xylose-responsive protein XylR and the corresponding promoter P_xylR_ has been developed in EcN, in which xylose can induce engineered bacteria to lyse and release the immune regulatory protein AvCystatin [100]. This system has the potential to be a disposable and recyclable candidate for treating gut inflammation.

**Table 2:** Summary of engineered bacteria using an artificial intervention system for IBD treatment.

Chassis	Inducer and induction system	Payload	Model	Refs
*Lactococcus lactis*	Xylose: XylR-P_xylT_	IL-10	TNBS-induced colitis	[99]
*Escherichia coli* Nissle 1917	Arabinose: AraC-P_araBAD_	Trefoil factors	DSS-induced colitis	[95]
*Escherichia coli* Nissle 1917	Blue light: OmpR-P_Dawn_	IL-10	DSS-induced colitis	[104]
*Escherichia coli* Nissle 1917	Blue light: OmpR-P_Dawn_	AG43 and TGF-β1	DSS-induced colitis	[105]
*Bacteroides ovatus*	Xylan: xylanase promoter	Human keratinocyte growth factor-2	DSS-induced colitis	[96]
*Bacteroides ovatus*	Xylan: xylanase promoter	Transforming growth factor β	DSS-induced colitis	[97]

Compared to chemical induction, optogenetics offers improved controllability, spatiotemporal specificity, and biosafety [[101], [102], [103]]. Cui *et al*. engineered a light-responsive EcN strain that expressed and secreted IL-10 [104]. This light-responsive EcN strain suppressed intestinal inflammation in ulcerative colitis mice and protected the intestinal mucosa against injury. Further, Cui *et al*. developed upconversion microgels (UCMs) that convert near-infrared light (NIR) into visible blue light to activate recombinant EcN [105]. This allowed for the secretion of the autotransporter 43 adhesin antigen (AG43) and precise spatiotemporal colonization in the intestinal tract. Live experiments demonstrated that colonizing bacteria effectively alleviated inflammation in mice with colitis. Optogenetics offers the advantages of spatiotemporal precision and the ability to control downstream functions with ease. However, its potential clinical applications are limited due to its restricted ability to penetrate deep tissues; for example, delivering light to the gut is more challenging than in a controlled laboratory setting. This limitation necessitates innovative solutions for light delivery, such as the development of implantable devices or the creation of light-activated compounds that can be triggered by internally generated light. Ultrasonography, with higher tissue penetration, may be an alternative strategy. Indeed, some studies have demonstrated the potential of ultrasound responsive-bacteria for tumor treatment [[106], [107], [108], [109]]. Moreover, light exposure required for optogenetic control could pose safety risks, such as tissue heating or DNA damage, especially during long-term applications. In conclusion, while optogenetics in bacteria offers exciting possibilities for transforming clinical approaches to disease treatment, significant technical, regulatory, and biological hurdles remain to be addressed.

## 3 Genetically engineered bacteria for the detection of IBD

Timely intervention and control of disease symptoms in patients with IBD rely heavily on early diagnosis. The development of whole-cell bacterial biosensors offers a distinctive solution for timely detection of biomarkers [110,111]. The bacteria engineered for IBD detection are summarized in Table 3. Typical whole-cell biosensors contain three modules: sensing (input), processing, and output. By finely adjusting and optimizing these modules, sensors can be endowed with sufficient sensitivity and outputs for disease detection [112]. During colitis, sulfate-reducing bacteria produce H_2_S, which is converted to thiosulfate by host enzymes to alleviate toxicity [113,114]. Furthermore, ROS generated by the host convert thiosulfate to tetrathionate [115]. Therefore, thiosulfate and tetrathionate levels may indicate colitis. *Salmonella typhimurium* uses the TtrR/TtrS two-component system (TCS) to sense tetrathionate as a terminal respiratory electron acceptor, providing growth advantages in the inflamed gut [116]. Riglar *et al*. coupled the TtrR/TtrS TCS with a toggle switch (CI/Cro) in NGF-1, a commensal mouse *E. coli* strain, to create *E. coli* strain PAS638 [117]. To minimize the burden and enhance stability, all components were integrated into the NGF-1 chromosome. PAS638, which utilizes the β-galactosidase gene as a reporter, can effectively be induced by tetrathionate (EC_50_: 0.38–0.85 μM; 95% confidence interval), and has been used to detect tetrathionate in two mouse models of inflammation for a period of six months. The TtrR/TtrS TCS is repressed by O_2_ and nitrate [118]. However, in the inflamed area, there is an increase in nitrate content [119], and a possibility of higher O_2_ levels in the region closer to the blood supply. Therefore, undesired cross-regulation of *S. typhimurium* TtrR/TtrS could potentially compromise its effectiveness in the gut. Daeffler *et al*. identified a TtrR/TtrS TCS from *Shewanella baltica* OS195 that was only minimally repressed by O_2_ and not repressed by nitrate in *E. coli* [120]. Further, they discovered a novel TCS named ThsS/ThsR in *Shewanella halifaxensis* HAW-EB4, which was the first known thiosulfate sensor [120]. The thiosulfate sensor, using sfGFP as a reporter, was transferred into an EcN strain. A method involving oral gavage and flow cytometric analysis of colon and fecal samples was then developed to show that colitis activates thiosulfate sensors in mice. Another gene-sensing circuit was developed to detect hydrogen polysulphide as an indirect method for detecting H_2_S [121]; however, it has not been tested in vivo.

**Table 3:** Summary of engineered bacteria for the detection of IBD.

Chassis	Target and sensor	Reporter	Model	Refs
*Escherichia coli* NGF-1	Tetrathionate: TtrR/TtrS-P_ttrSR_	LacZ	Inflammation induced by *S. typhimurium*	[117]
*Escherichia coli* Nissle 1917	Tetrathionate: TtrR/TtrS-P_ttrB_ and Thiosulfate: ThsS/ThsR-P_phsA_	sfGFP	DSS-induced colitis	[95]
*Escherichia coli* Nissle 1917	Nitrate: NarX/NarL-P_yeaR_	sfGFP	DSS-induced colitis	[127]
*Escherichia coli* Nissle 1917	Heme: HrtR/ChuA-P_hrtR_	LuxCDABE	DSS-induced colitis	[129]
*Escherichia coli* Nissle 1917	pH: SO_4387-SO_4388_REC_-PsdR_DBD_	sfGFP	TNFΔARE mice	[130]
*Escherichia coli* Nissle 1917	Calprotectin: Zur-P_ykgMO_	sfGFP and LuxCDABE	DSS-induced colitis	[134,135]
*Escherichia coli* Nissle 1917	NO: NorR-P_norV_, H_2_O_2_: OxyR-P_oxyS_, Tetrathionate: TtrR/TtrS-P_ttrB_ and Thiosulfate: ThsS/ThsR-P_phsA_	sfGFP and LuxCDABE	DSS-induced colitis	[142]
*Escherichia coli* TOP10	NO: NorR-P_norV_	CFP	Mouse intestinal explants	[126]

Gut inflammation can upregulate the expression of inducible nitric oxide synthase (iNOS), resulting in elevated production of nitric oxide (NO) [122,123]. Furthermore, NO can be readily converted into nitrate [124]. Studies have demonstrated an increase in both nitrate and NO levels in mice with colitis [119,125]. Archer *et al*. utilized the enhancer binding protein NorR to develop an NO sensor owing to its high specificity for NO [126]. When NO binds to NorR, the GAF domain releases its repression on the AAA+ domain, enabling it to bind to σ^54^ and initiate the transcription of P_norV_. Subsequently, a hybrid circuit was created using an NO sensor and DNA recombinase by placing the *fimE* gene under the control of P_norV_. When the circuit detects the presence of NO, the bacteria switch from expressing a yellow protein to a cyan protein. Woo *et al*. employed the NarX-NarL TCS in an EcN strain to create a bacterial biosensor that responds to nitrate [127]. Using this bacterial sensor, increased nitrate levels were successfully detected in mice with DSS-induced colitis.

Several bacterial sensors have been developed to detect biomarkers associated with complications of intestinal inflammation. Detection of fecal occult blood plays a critical role in the early diagnosis and treatment of gut inflammation [128]. A split-*lux* cassette was coupled with a toggle switch in the EcN strain to create a bacterial sensor (YES601) for highly sensitive detection of a blood marker heme with a detection limit of 0.019 μM (LogEC_50_: 0.0718 μM) [129]. YES601 contains several key components, including a heme-responsive transcriptional repressor, HrtR, the heme transporter ChuA, a genetic toggle switch, and a set of bioluminescence systems. These elements work together to enable the detection of heme and subsequent activation of bioluminescence in response to its presence. The YES601 bacterial sensor was able to detect fecal occult blood in DSS-induced mice as early as the second day, whereas the commercial chemical method only yielded results in the third-day sample [129]. Tissue inflammation induces metabolic changes, leading to extracellular acidosis. Cartwright *et al*. constructed an engineered bacterial sensor capable of sensing pH changes and emitting light under acidic conditions to detect acidosis caused by intestinal inflammation [130]. Various diseases or environmental factors, such as colorectal cancer, can lead to fecal occult blood and changes in intestinal pH. Therefore, while fecal occult blood and intestinal pH can serve as biomarkers for the preliminary detection of IBD, they cannot be used as definitive criteria for its diagnosis.

The potential of most of these compounds as molecules involved in human IBD requires further investigation. Calprotectin, released by neutrophils, is widely regarded as the clinical gold standard biomarker for assessing gut inflammation [[131], [132], [133]]. It is commonly measured in fecal samples to evaluate the presence and severity of gastrointestinal tract inflammation. Xia *et al*. recently discovered a series of calprotectin-responsive promoters in the EcN strain, which are associated with the cellular regulation of zinc ion (Zn), and the ykgMO promoter in EcN strains was used to create an engineered bacterial sensor for detecting calprotectin, achieving a lower limit of detection of 25 µg/g [134]. Using murine models of colitis, this bacterial sensor was demonstrated to be activated *in vivo* upon oral administration. Furthermore, this bacterial sensor can distinguish between patients with active IBD and those in remission or without IBD by analyzing patient stool samples. In an independent study, Zhu *et al*. designed a bacterial sensor that detects calprotectin through the ykgMO promoter and developed a self-regulated therapy for colitis based on this sensor [135]. It is worth noting that the ykgMO promoter cannot directly sense calprotectin, but rather the zinc restriction conditions caused by calprotectin [136,137]. *E. coli* utilizes an evolutionarily conserved mechanism to regulate gene expression in response to zinc limitation. This mechanism relies on the transcription factor Zur, which controls the expression of the genes *ykgM* and *ykgO*. Zur represses transcription when zinc concentrations are sufficient but becomes inactive when intracellular zinc levels fall below a critical threshold [[138], [139], [140]]. Calprotectin possesses a strong chelating ability for metal ions, such as zinc, which can be leveraged to create conditions that restrict zinc availability and activate the expression of downstream genes in Zur [136,137]. The properties of calprotectin make the Zur and ykgMO promoters attractive candidates for engineering calprotectin biosensors.

Combining an engineered bacterial sensor with ultralow-power microelectronics has the potential to enable *in situ* detection of gastrointestinal biomolecules that are associated with both health and disease. This approach offers real-time monitoring and analysis of biomarkers within the gastrointestinal tract, providing valuable insights into the dynamic changes occurring in the gut and potentially facilitating early detection and intervention in various health conditions. Mimee *et al*. described an ingestible micro-bioelectronic device that combines a heme-responsive bacterial sensor with ultralow-power microelectronics for real-time monitoring of gastrointestinal bleeding events [141]. Recently, Inda-Webb *et al*. further upgraded the electronic capsule and combined it with bacterial sensors to obtain a real-time disease biomarker monitoring system. This integrated system monitors various inflammatory markers in the gut, including NO, hydrogen peroxide, thiosulfate, and tetrathionate, in both mouse and pig models of inflammation [142]. Although combining engineered bacterial sensors with ultralow-power microelectronic technology has certain application potential, it is still necessary to evaluate their usage costs and patient acceptability as a minimally invasive detection method.

Capturing and recording dynamic changes in the gut poses a significant challenge [143,144]. Schmidt *et al*. developed a CRISPR-based recording method called Record-seq that allows the capture and conversion of intracellular RNAs into DNA [145,146]. This innovative approach enables DNA-based storage of transcriptional information in *E. coli*. Schmidt *et al*. used this method to capture transcriptional alterations occurring in *E. coli* as they traverse the intestines. In a colitis model, bacterial recorders recorded transcriptional changes indicative of decreased anaerobic metabolism, an activated stringent response, and elevated oxidative and membrane stress [147].

## 4 Self-tunable bacteria for closed-loop therapy of IBD

Closed-loop therapy refers to a technology designed to automatically monitor and adjust treatment based on a patient's real-time health data. This system incorporates a feedback loop which allows for continuous control of treatment variables such as medication dosage without manual intervention. For bacterial therapy, constitutive or artificial intervention systems pose difficulties in accurately controlling the timing and dosage of drug release, leading to ineffective treatment or overdosing. In contrast, engineered bacteria with tuned feedback control loops can sense specific disease biomarkers and release therapeutic payloads at lesion sites (closed-loop therapy) [31,32]. Intelligent closed-loop therapy has been developed for various chassis (bacterial or mammalian) cells and applied to the treatment of a range of diseases, including IBD (Table 4), diabetes, and cancer [31,32].

**Table 4:** Summary of self-tunable bacteria for the closed-loop therapeutic of IBD.

Chassis	Target	Payload	Model	Refs
*Lactococcus lactis*	Stress (pH, heat-shock, or bile salts)	IL-10	TNBS-induced colitis	[148]
*Escherichia coli* Nissle 1917	Tetrathionate	Microcin H47	*S. typhimurium*	[149]
*Escherichia coli* Nissle 1917	NO	IFNL1	*In vitro* IBD model	[150]
*Escherichia coli* Nissle 1917	NO	CheZ-YabQ and GM-CSF	*In vitro* Crohn's disease model	[151]
*Escherichia coli* Nissle 1917	Thiosulfate	AvCystatin	DSS-induced colitis	[152]
*Saccharomyces cerevisiae*	eATP	ATP-degrading enzyme apyrase	DSS-induced colitis and TNBS-induced colitis	[153]
*Escherichia coli* MG1655	Tetrathionate	TtrBCA and CheZ	*In vitro* simulation	[154]

Benbouziane *et al*. developed a stress-inducible controlled expression system in *L. lactis*, which enables the targeted delivery of IL-10 *in situ* [148]. Palmer *et al*. developed an EcN strain that responds to tetrathionate to produce microcin H47, an antibacterial peptide that inhibits the growth of *Salmonella in vitro* [149,155]. Recently, Chua *et al*. genetically engineered EcN to produce and secrete interferon lambda 1 (IFNL1) by sensing NO which exhibited anti-inflammatory effects in an *in vitro* IBD model [150].

Genetically modified bacteria that can recognize disease biomarkers and colonize specific areas are promising candidates for engineered bacterial therapeutics. McKay *et al*. constructed an NO-responsive system in *E. coli* to produce CheZ-YabQ and confer pseudotaxis [151]. Using this system, the *E. coli* strain was capable of sensing and orienting its movement towards NO. Furthermore, the expression of granulocyte-macrophage colony stimulating factor (GM-CSF) was incorporated into the pseudotaxis system as a treatment for IBD. However, owing to its gaseous nature, NO can diffuse extensively into the intestinal environment, resulting in imprecise targeting. Therefore, the identification of a biomarker of gut inflammation with a limited diffusion range is crucial for enhancing the targeting accuracy. Recently, an engineered *E. coli* strain was reconstructed that can orient tetrathionate chemotaxis [154]. This strategy enables engineered bacteria to respire tetrathionate through the tetrathionate reductase gene cluster (*ttrBCA*), allowing the engineered bacteria to compete with *S. typhimurium* in the inflamed intestine. Although the concept of disease marker tropism is appealing, it remains unclear whether such adaptations can effectively promote the recruitment of bacteria to areas of inflammation in the intestinal environment owing to intestinal contractions and well-known complex factors.

**Fig. 2:**
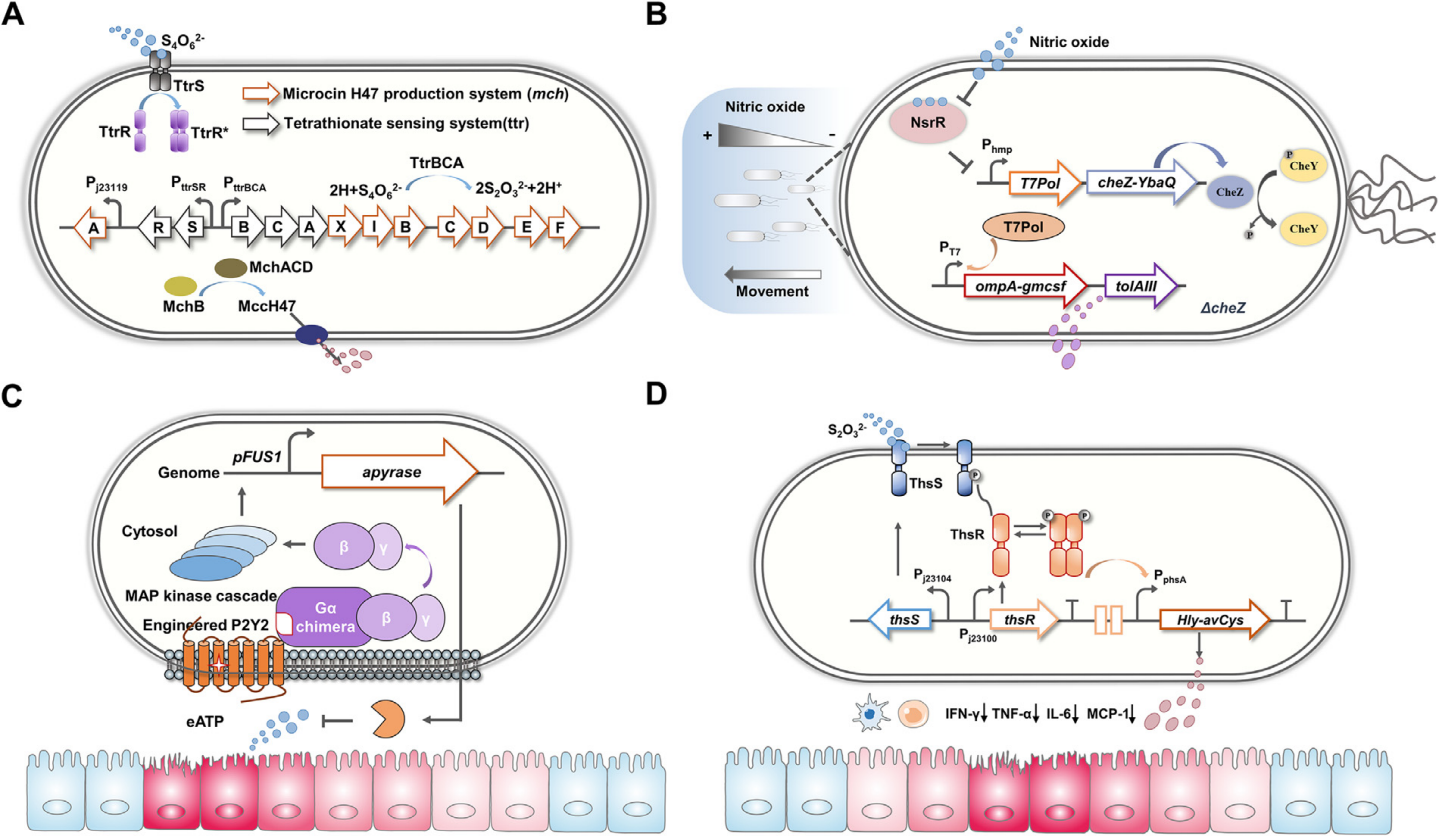
Schematic diagrams of the self-tunable bacterial design for closed-loop treatment of inflammatory bowel disease. (A) In the presence of tetrathionate, the engineered strain, equipped with a plasmid-based system containing the *ttrRSBCA* and *mchAXIBCDEF* gene clusters, can produce MccH47. Expression of the tetrathionate reductase gene cluster *ttrBCA* and MccH47 expression gene cluster *mchAXIBCDEF* is positively regulated by the TtrS/TtrR TCS [149]. (B) In the engineered bacteria, the hmp promoter, controlled by nitric oxide regulatory protein NsrR, on one plasmid is responsible for generating the T7 RNA polymerase, which subsequently drives expression under the P_T7_ on a second plasmid. This mechanism enables the overexpression of granulocyte-macrophage colony stimulating factor while maintaining natural levels of expression for CheZ [151]. (C) In the self-tunable engineered yeast system, the engineered human P2Y2 receptor detects eATP, which subsequently activates the pFUS1 promoter. Activation of the receptor by eATP results in the expression and secretion of apyrase by the yeast [153]. (D) The engineered EcN strain detects thiosulfate through the ThsS/ThsR TCS and subsequently activates the *phsA* promoter, which results in the expression and secretion of AvCystatin [152].

Several self-tunable bacterial therapies have been shown to sense inflammatory markers and release the corresponding payloads to effectively ameliorate colitis in mice. The production of extracellular adenosine triphosphate (eATP) by the commensal microbiota and host cells triggers purinergic signaling, leading to the promotion of intestinal inflammation and pathology [85,86]. Scott *et al*. developed an engineered yeast strain that can sense and degrade eATP to treat IBD [153]. This self-modulatory yeast strain contains a sensing element (human P2Y2 receptor) and a functional element (apyrase, an ATP-degrading enzyme). These self-tunable yeast probiotics were demonstrated to effectively suppress intestinal inflammation in mice with colitis and reduce intestinal fibrosis and dysbiosis. Moreover, thiosulfate-responsive engineered EcN was demonstrated to self-regulate the expression and release of the immunomodulatory agent AvCystatin, effectively improving colitis in mice [152]. A set of schematic diagrams of the self-tunable bacterial design for the closed-loop treatment of IBD is shown in Fig. 2.

## 5 Intelligent live bacterial therapies with integrated diagnosis and treatment

Smart multifunctional sense-and-respond genetic circuits have been developed in bacterial chassis for integrated diagnosis and treatment of diseases. An engineered intelligent EcN strain, i-ROBOT (intelligent Responsive Bacteria for Diagnosis and Therapy), has been developed based on three key functions (fluorescence reporting, a base-editing system, and drug secretion) [152,156]. This intelligent strain can report the presence of disease markers and record events of interest such as the emergence of thiosulfate in the gut. This is achieved by translating biomarker stimulation into permanent single-nucleotide alterations in genomic DNA, leading to the translational activation of functional proteins (such as LacZ). This information can be passed on to subsequent generations without loss. Additionally, i-ROBOT features a self-tunable drug-secretion system induced by the inflammatory marker thiosulfate. Oral administration of i-ROBOT to mice with colitis generated molecular recordings and fluorescence signals in processed fecal and colon samples and effectively ameliorated the disease. Recently, Zhu *et al*. developed an engineered probiotic that could diagnose and treat IBD by sensing calprotectin [135]. Gurbatri *et al*. engineered an EcN strain to simultaneously detect and treat colorectal neoplasia [157]. From our perspective, multifunctional intelligent live bacterial therapies show great potential for advancing personalized medicine into the next generation owing to their ability to provide precise diagnosis and treatment.

## 6 Concluding remarks and future perspectives

The use of bacteria for the site-directed delivery of anti-inflammatory payloads is an extremely appealing strategy for the treatment of IBD. Moreover, live bacterial medicines can be programmed to recognize and respond to specific disease signals, thereby reporting the disease status and providing therapeutic payloads. This is an exciting area of research that can target therapy in the affected areas of the body, maximize efficacy, and minimize potential side effects. Nonetheless, it is crucial to evaluate the safety, efficacy, biocontainment, and stability of live bacterial therapies for IBD before clinical translation, as these factors determine the ultimate success of the treatment.

### 6.1 Biosafety

Biosafety is a primary concern in the clinical application of live-bacterial therapies. The choice of a suitable chassis or host organism plays a crucial role in ensuring the safety and effectiveness of live bacterial therapies. Several bacterial strains such as *L. lactis, Lactobacillus plantarum, Lactobacillus bulgaricus*, and EcN have been designated as GRAS and have demonstrated positive effects in improving IBD [[158], [159], [160], [161]]. Although human clinical trials of engineered bacterial medicines have demonstrated their safety, many treatment results have been disappointing, mainly due to the inability of the chassis to survive long enough to express payloads [40]. Therefore, certain strains abundant in the gut microbiome (such as *Bacteroides* spp.) have been suggested as potential transgenic chassis strains [162]. Nevertheless, engineering of these species poses significant challenges [163,164]. Recently, Russell *et al*. presented a proof-of-concept study that engineered a native *E. coli* strain from the host to express a transgene of interest (such as bile salt hydrolase, a prokaryotic bile acid deconjugation enzyme, or IL-10) to modify the host phenotype and treat diseases [165]. While the novelty and potential of engineered native *E. coli* have been demonstrated, there are still significant limitations that need to be addressed: (1) Biosafety: Despite the maximum adaptation of native bacteria to the host luminal environment, there is still a need for significant evidence to prove whether the long-term implantation of engineered natural bacteria will cause unpredictable disturbances to the native bacterial community or the host physiological environment. (2) Genetic modifications: Most native *E. coli* and other bacteria are resistant to genetic manipulation, and more effective methods for engineering native bacteria need to be developed. (3) Universality: Extensive research is required to determine whether engineered native bacteria can successfully colonize individuals other than the source host, as customizing engineered native bacteria for different individuals is time-consuming and labor-intensive.

Horizontal gene transfer (HGT) refers to the transfer of genetic material between different organisms, including bacteria [166,167]. In the context of live bacterial therapy, HGT can occur between administered bacteria and native bacteria present in the host microbiome [168]. If the transferred genes contain virulence factors or antibiotic resistance genes, they pose safety concerns and compromise the effectiveness of the therapy. Therefore, integrating functional elements into the bacterial genome, or combining biological protective measures, can help alleviate the risks associated with HGT.

### 6.2 Accuracy

Intestinal inflammation is a complex process involving multiple disease markers, which makes it challenging to rely on a single marker for accurate diagnosis and treatment. To address this issue, genetically engineered bacteria with multi-input biological computation capabilities can provide precise regulation and spatiotemporal control of therapeutic payloads [[169], [170], [171], [172], [173]]. AND logic can enhance the specificity of engineered bacteria in response to inflammatory signals, whereas OR logic can help distinguish between different stages of inflammation [174]. NOT logic ensures additional safety [175]. Although previous studies have demonstrated the feasibility of using dual-input engineered bacteria to detect inflammatory biomarkers, these systems are yet to be tested in actual gut environments [127,175]. When designing biocomputing circuits, it is important to consider the chronological order of the appearance of inflammatory markers rather than simply assembling sensors for each signal. For example, an AND logic operation should be employed when inflammatory markers appear simultaneously, whereas an OR gate should be used when the markers appear at different times.

In clinical practice, indicators like calprotectin, C-reactive protein, and inflammatory cytokines (such as TNF-α, IL-6, and IFN-γ) are crucial for diagnosing human IBD. Unfortunately, owing to the multilayered membrane structure of bacteria, the direct detection of protein biomarkers poses challenges for bacterial sensors. One potential solution is to reconstruct bacterial two-/multicomponent systems using nanoantibodies [176,177]. Although this approach is promising, it requires extensive experimentation and practical evidence to determine its effectiveness.

The accuracy of IBD diagnosis and treatment relies heavily on the sensitivity and specificity of engineered bacterial sensors for detecting disease markers. Although numerous synthetic biology strategies have been developed to enhance the performance of these sensors [112,[178], [179], [180], [181]], it remains unclear whether they can function robustly in complex intestinal environments without sufficient data. The development of a human gut chip model provides a simple and effective platform for high-throughput screening of a vast collection of bacterial sensor candidates [182]. This approach may accelerate the identification of promising sensor candidates and ultimately improve the precision of IBD diagnosis and treatment.

### 6.3 Delivery

The efficient delivery of bacteria to intestinal lesions is a pressing challenge. Factors such as stomach acids, digestive enzymes, and bile salts can reduce the viability of bacteria, thereby limiting the number of live probiotics that reach the gastrointestinal targets. In addition, the continuous flow within the gastrointestinal tract and pressure from the native bacterial community can result in low oral bioavailability and limited intestinal colonization. To address these barriers, researchers have developed various bacterial encapsulation delivery strategies, including hydrogels, chemical surface coatings, and surface chemical conjugation [[183], [184], [185]]. These include hydrogel encapsulation, which involves embedding bacteria within a hydrogel matrix to provide protection; surface modification of bacteria to enhance their stability and targeting capabilities; and bioinspired strategies such as self-coating with biofilms to mimic natural protective mechanisms and improve the viability and functionality of encapsulated bacteria. However, it is important to note that despite their potential to advance the clinical application of live bacterial therapy, bacterial encapsulation systems may also hinder the release of therapeutic proteins and reduce efficacy. This issue must be carefully considered for next-generation bacterial encapsulation systems.

**Table 5:** Summary of bacterial delivery systems.

Delivery systems	Brief introduction	Characteristics
Hydrogels	Hydrogels are polymeric materials with a network-like structure that can hold a large amount of water while maintaining their form.	High water content: Hydrogels can contain over 90% water, which is retained within their polymer networks. Softness and flexibility: due to their significant water content, hydrogels are soft and flexible, closely mimicking the mechanical properties of natural tissues. Biocompatibility: many hydrogels are biocompatible, making them suitable for delivery systems. Responsive to environmental stimuli: hydrogels can be engineered to be sensitive to changes in temperature, pH, or the presence of specific metabolites or enzymes.
Chemical Surface Coating	Chemical surface coating of bacteria involves modifying the exterior of bacterial cells using chemicals to change their surface properties to enhance the functionality and usability of bacteria.	Polymer coatings: applying synthetic or natural polymers that can encapsulate the bacteria for protection or to facilitate attachment to surfaces. Lipid coatings: coatings that modify the lipids on the bacterial surface to alter their interaction with the human immune system or other cells.
Surface Chemical Conjugation	Bacterial surface chemical conjugation refers to the process of molecular chemical modification on the surface of bacterial cells. This process primarily utilizes reactive groups such as thiols and amino groups present on the bacterial cell wall as anchoring sites for conjugation.	Functionalization: conjugating molecules like proteins, peptides, or synthetic compounds to bacteria can introduce new functions or enhance existing ones. Targeting and recognition: modified bacteria can be designed to target specific tissues, cells, or pathogens. Immobilization: conjugated molecules can facilitate the attachment of bacteria to different substrates.

In summary, genetically engineered live bacterial therapies hold significant potential for clinical applications in the treatment and detection of intestinal diseases, such as IBD. However, before these therapies can be widely used, it is crucial to address concerns regarding their biosafety, efficacy, and efficient delivery.

## CRediT authorship contribution statement

**Zhen-Ping Zou:** Writing – original draft, Conceptualization. **Xiao-Peng Zhang:** Writing – original draft. **Qian Zhang:** Writing – review & editing. **Bin-Cheng Yin:** Writing – review & editing. **Ying Zhou:** Writing – review & editing, Writing – original draft, Funding acquisition. **Bang-Ce Ye:** Writing – review & editing, Conceptualization.

## Declaration of competing interest

The authors declare that they have no known competing financial interests or personal relationships that could have appeared to influence the work reported in this paper.
